# A New Abietene Diterpene and Other Constituents from *Kaempferia angustifolia* Rosc.

**DOI:** 10.3390/molecules16043018

**Published:** 2011-04-07

**Authors:** Sook Wah Tang, Mohd Aspollah Sukari, Mawardi Rahmani, Nordin Hj. Lajis, Abdul Manaf Ali

**Affiliations:** 1 Department of Chemistry, Faculty of Science, Universiti Putra Malaysia, 43400 UPM Serdang, Selangor, Malaysia; 2 Universiti Sultan Zainal Abidin, Kampus Kota, Jalan Sultan Mahmud, 20400 Kuala Terengganu, Malaysia

**Keywords:** *Kaempferia angustifolia*, abietene diterpene, kaempfolienol, zeylenol, cytotoxic

## Abstract

A new abietene diterpene, kaempfolienol (5*S*,6*S*,7*S*,9*S*,10*S*,11*R*,13*S*-abiet-8(14)-enepenta-6,7,9,11,13-ol, **1**), was isolated from a rhizome extract of *Kaempferia angustifolia* Rosc. along with the known compounds crotepoxide, boesenboxide, zeylenol, 2′-hydroxy-4,4′,6′-trimethoxychalcone, (24*S*)-24-methyl-5α-lanosta-9(11),25-dien-3β-ol, β-sitosterol and β-sitosterol-3-*O*-β-D-glucopyranoside. The structures of all compounds were elucidated on the basis of mass spectroscopic and NMR data. Zeylenol (**2**), the major constituent of the plant, was derivatized into diacetate, triacetate and epoxide derivatives through standard organic reactions. The cytotoxic activity of compounds **1**, **2** and the zeylenol derivatives was evaluated against the HL-60, MCF-7, HT-29 and HeLa cell lines.

## 1. Introduction

*Kaempferia angustifolia*, which is also known as *Kunci pepet*, *Kunci menir* or *Kunci kunot* can be found growing wild in forests of Western and Central Java of Indonesia and in parts of Thailand. The pleasant-smelling plant is usually used as a remedy for colds, stomach ache and dysentery, while its rhizomes are used for coughs and as a masticatory [1]. Our group has previously reported on the chemical constituents [1], essential oil characterization [2] and the larvicidal activity of *Kaempferia angustifolia* against *Aedes aegypti* larvae [3]. Besides oxygenated cyclohexane derivatives [4], pimarane diterpenes [5], phenylpropanoids [6] and flavonoids [7] have also been isolated from other *Kaempferia* species. This paper reports our recent isolation of a new abietene diterpene, kaempfolienol (**1**), along with chalcone, triterpene, glycoside and other cyclohexane derivatives from the rhizome of *Kaempferia angustifolia* and the cytotoxic properties of selected compounds. Several derivatives of the major component zeylenol were also prepared to evaluate the potential enhancement of its cytotoxic activity. 

## 2. Results and Discussion

Phytochemical investigation on the rhizomes of *Kaempferia angustifolia* Rosc. from Indonesia has resulted in the isolation of new abietene diterpene kaempfolienol and seven other known compounds. The structures of crotepoxide [1], boesenboxide [1], 2′-hydroxy-4,4′,6′-trimethoxychalcone [1], (+)-zeylenol [4], (24*S*)-24-methyl-5α-lanosta-9(11),25-dien-3β-ol [8], β-sitosterol [9] and β-sitosterol-3-*O*-β-D-glucopyranoside [10] were elucidated based on comparison of their physical and spectral data with reported literature values. 

**Figure 1:**
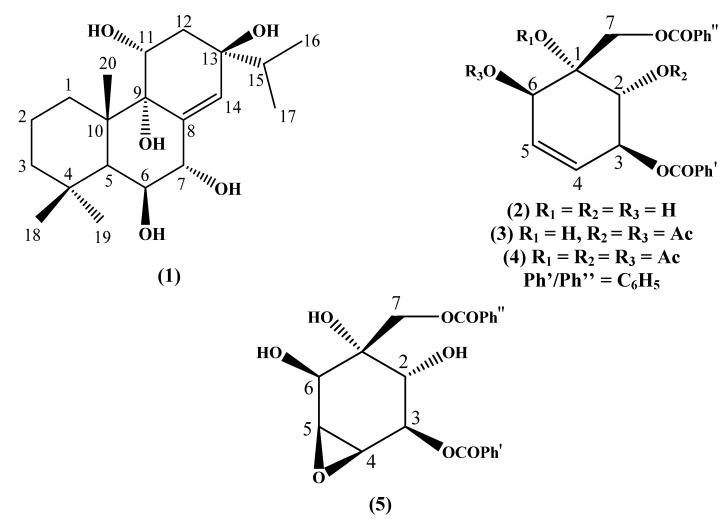
Structures of compounds.

Compound **1**, kaempfolienol (Figure 1), was isolated from the chloroform extract of *Kaempferia angustifolia* rhizome as colourless needle-shaped crystals with a melting point of 278-280 °C. It has an optical rotation value of [α]_D_ = + 1.8 ° (*c* 0.13, MeOH). A sodium adduct molecular ion at *m/z* 377.2746 (calcd. 377.2304) [M+Na]^+^ corresponding to the molecular formula C_20_H_34_O_5_Na was observed in the HRESIMS spectrum, requiring four unsaturation equivalents. The absorptions at 3,438 cm^-1^ and 1,018 cm^-1^ in the IR spectrum were attributed to hydroxyl group and C-O, respectively, whereas a weak absorption band at 1,628 cm^-1^ was due to olefinic C=C stretching. These data indicated that compound **1** is an unsaturated alcohol derivative. The ^1^H-NMR spectrum was integrated for 29 protons. The presence of five methyl signals at δ 1.27, 1.21, 1.02, 0.96 and 0.93, of which the latter two, due to an isopropyl group, resonated as a doublet with *J* = 7.0 Hz, indicating that compound **1** is an abietane-type diterpene.

**Figure 2:**
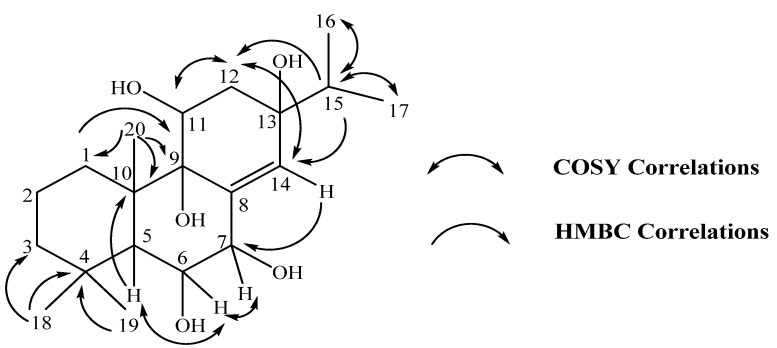
COSY and HMBC correlations of compound **1**.

In addition, an olefinic proton at δ 5.78 (s) and three methine protons at δ 4.35 (*dd*, *J* = 11.3, 4.9 Hz), 4.18 (*t*, *J* = 2.8 Hz) and δ 3.89 (*d*, *J* = 2.8 Hz) in the lower field region were also observed. The proton at δ 4.18 showed a cross peak with the signal at δ 3.89 in the COSY spectrum, revealing that they were linked to adjacent carbons. The occurrence of an isopropyl group was also supported by the COSY correlations between two methyls at δ 0.96 (H-16) and 0.93 (H-17) with the methine proton (H-15) at δ 1.71 (*m*) (Figure 2). 

From the ^13^C-NMR spectrum, 19 carbon resonances which corresponded to six methine, four methylene, five methyl and four quartenary carbons from DEPT analysis were observed. One of the carbon signals at δ 45.0 was possibly due to overlapping of two carbon resonances since the intensity of the peak was about one-fold higher than the rest and the assignments remained to be clarified. A pair of olefinic carbons was observed at δ 139.9 (q) and 135.8 (methine), while crowded signals in the high-field region δ 15-50 were typical for a terpene skeletons.

The HMQC spectrum showed carbons and protons connected by one bond whilst HMBC correlations determined two and three-bond carbon-proton couplings (Figure 2). Two quartenary methyls at δ 1.27 (H-18) and 1.02 (H-19) were correlated to δ 35.0 (C-4) in the HMBC spectrum; the former signal also showed cross peak with methylene carbon at δ 45.0 (C-3), while the latter exhibited a ^3^*J* correlation with the methine carbon at δ 44.3 (C-5).

Furthermore, H-5 (δ 1.96) displayed a correlation to another quartenary methyl (C-20: δ 21.2), and its protons (H-20: δ 1.21) were in turn correlated to the δ 37.1 methylene carbon (C-1), oxygenated quartenary carbon δ 78.0 (C-9) and the signal at δ 45.0 in the HMBC spectrum. Therefore, the carbon resonance at δ 45.0 was assigned to the quartenary carbon C-10 in addition to C-3. Correlations between H-5 and δ 4.18 (H-6) and between H-6 and δ 3.89 (H-7) were demonstrated in the COSY spectrum, thus revealing the connectivities of C5-C7. 

Methyl protons (H-16 and H-17) and the methine proton H-15 from the isopropyl fragment were correlated to the oxygenated quartenary carbon at δ 73.3 (C-13). H-15 also demonstrated long-range correlations to the methylene carbon δ 37.6 (C-12) and the olefinic carbon δ 135.8 (C-14). Again, the elucidation of structure was substantiated by COSY correlations between the olefinic proton H-14 at δ 5.78 and H-12 at δ 1.61 (via a *w*-coupling) which also displayed a cross peak with the oxygenated methine proton H-11 (δ 4.35). The connectivities shown from the evidences mentioned above revealed the double-bond at C-8 (δ 139.9) and C-14 (δ 135.8) and the placement of isopropyl group at C-13. 

Additional HMBC correlations of H-11/C-10, H-14/C-7 (δ 81.2) and H-1 (δ 1.54)/C-9 (78.0) established the linkage between three cyclic rings. Proton signals for hydroxyl groups were not observed, but the placement of hydroxyl groups at C-6, C-7, C-9, C-11 and C-13 was reasonable since the respective carbons resonated in a more deshielded region (δ 65 – 81) and conform to the type of carbons in DEPT spectrum. Consequently, compound **1** was identified as abiet-8(14)-enepenta-6,7,9,11,13-ol and named kaempfolienol. The NMR spectral data of this compound are summarized in Table 1. 

**Table 1:** ^1^H-NMR (500 MHz) and ^13^C-NMR (125 MHz) spectral data of kaempfolienol (**1**) (CD_3_OD).

Position	^1^H-NMR δ (multiplicity, *J* in Hz)	COSY	^13^C -NMR (δ)	HMBC	
				^2^ *J*	^3^ *J*
1	1.55 (*m*)	-	37.1	C-2	C-9
	1.76 (*dd*, 13.1, 3.4)				
2	1.46 (*dt*, 13.1, 3.4)	-	20.0	-	-
	1.66 (*m*)				
3	1.25 (br *d*, 3.4)	-	45.0	-	C-1
	1.33 (br *d*, 11.3)				
4	-	-	35.0	-	-
5	1.96 (*d*, 2.8)	H-6	44.3	C-6, C-4,C-10	C-9, C-20,C-18, C-3
6	4.18 (*t*, 2.8)	H-5, H-7	73.1	C-7	C-10, C-8
7	3.89 (*d*, 2.8)	H-6	81.2	C-6, C-8	C-5, C-9, C-14
8	-	-	139.9	-	-
9	-	-	78.0	-	-
10	-	-	45.0	-	-
11	4.35 (*dd*, 4.9, 11.3)	H-12	65.4	C-12	C-10
12	1.61 (*dd*, 11.3, 1.5)	H-11	37.6	C-13, C-11	C-14, C-9, C-15
	1.63 (*dd*, 4.9, 1.5)				
13	-	-	73.3	-	-
14	5.78 (*s*)	H-12	135.8	C-13	C-7, C-9, C-12, C-15
15	1.71 (*m*)	H-16, H-17	39.3	C-13, C-16	C-12, C-14
16	0.96 (*d*, 7.0)	H-15	16.9	C-15	C-13, C-17
17	0.93 (*d*, 7.0)	H-15	17.6	C-15	C-13, C-16
18	1.27 (*s*)	-	25.0	C-4	C-3, C-19
19	1.02 (*s*)	-	34.3	C-4	C-18, C-5, C-3
20	1.21 (*s*)	-	21.2	C-10	C-9, C-1

The less clear stereochemical aspects of **1**, such as the ambiguity around the stereochemistry of chiral centres, led us to perform an X-ray diffraction analysis of the compound. The single crystal XRD data has been deposited with the Cambridge Crystallographic Data Centre (CCDC) [11]. The structure of **1** was thus confirmed as 5*S*,6*S*,7*S*,9*S*,10S,11*R*,13*S*-abiet-8(14)-enepenta-6,7,9,11,13-ol, and the compound was named as kaempfolienol. Abietane diterpenes are rarely described from *Kaempferia* species and the Zingiberaceae family. To date, the only mention found is isolation of abieta-8,11,13-trien-11-ol from *Kaempferia atrovirens* reported in a dissertation [12] and hence these findings contribute to the chemotaxonomic significance.

### 2.1. Derivatives of Zeylenol

Zeylenol (**2**), an oxygenated cyclohexene derivative, was isolated as the major compound from the chloroform extract of the plant. The synthesis of zeylenol derivatives has been reported previously [13] but some of the ^13^C-NMR spectral data have never been reported and their cytotoxic properties are yet to be studied. 

More efficient synthesis methods were implemented for the structural-modification of zeylenol (**2**) to give zeylenol diacetate (**3**) and zeylenol triacetate (**4**) in high yield. A higher yield (99.3%) for conversion of zeylenol (**2**) to derivative **3** was accomplished by using same reagents as in the literature [13], albeit with shorter reaction duration (2 hours) as compared to the overnight stirring used in the reference. The use of acetic anhydride under base (pyridine)-catalyzed conditions at reflux led to the formation of single product **4** (77.5% of yield), whereas a mixture of **3** (33%) and **4** (31%) was obtained by employing 4-dimethylaminopyridine and stirring at RT overnight [13]. 

The formation of diacetate derivative **3** (Figure 1) could be evidenced from two singlet peaks at δ 2.09 (2-OCOCH_3_) and 2.12 (6-OCOCH_3_) in the ^1^H-NMR spectrum. In addition, the signals for acetoxyl methyls [δ 20.9 (2-OCOCH_3_) and 20.8 (6-OCOCH_3_)] and the corresponding carbonyl carbons (δ 170.3 and 169.7) could also be observed in the ^13^C-NMR spectrum. 

The ^1^H-NMR spectrum of zeylenol triacetate (**4**, Figure 1) is similar to that of zeylenol (**2**), except for the presence of three extra singlet peaks at δ 1.96, 2.03 and 2.13 which can be assigned to the acetoxy methyls 2-OCOCH_3_, 6-OCOCH_3_ and 1-OCOCH_3_, respectively. The oxymethine protons H-2, H-3 and H-6 appeared more deshieded, whereas the inequivalent methylene protons of H-7 resonated at lower field upon acetylation compared to the starting material **2**. Besides, the formation of triacetoxy derivative could be evidenced from ^13^C-NMR shifts by having three methyl signals at δ 20.7 (2-OCOCH_3_), 20.8 (6-OCOCH_3_) and 21.6 (1-OCOCH_3_) as well as three carbonyl groups at δ 169.2 (1-OCOCH_3_), 169.5 (6-OCOCH_3_) and 169.9 (2-OCOCH_3_). 

As for the elucidation of zeylenol epoxide (**5**, Figure 1), the signals at δ_H_ 5.81 (δ_C_ 127.8) and δ_H_ 5.94 (δ_C_ 131.3) corresponding to the olefinic group of zeylenol (**2**), were no longer present. Instead, two doublet of doublet peaks were observed at the higher field region at δ 3.79 (H-4) and 3.67 (H-5) in the ^1^H-NMR spectrum. In the ^13^C-NMR spectrum, two additional methine carbon resonances at δ 57.0 (C-4) and 54.8 (C-5) were observed. Furthermore, ^3^*J* correlations between H-4 and C-2 as well as H-5 versus C-1 in HMBC spectrum proved the assignments of C-4 and C-5. The ^13^C-NMR spectral data of compounds **3** and **5** are reported for the first time in this paper.

### 2.2. Cytotoxic Activity of Compounds

The cytotoxic activities of compounds **1-5** were evaluated against HL-60 (human promyelocytic leukemia), MCF-7 (human breast cancer), HT-29 (human colon cancer) and HeLa (human cervical cancer) cell lines and the results are summarized in Table 2. Kaempfolienol (**1**) demonstrated moderate inhibition against HL-60 and MCF-7 cell lines with IC_50_ values 24.22 ± 0.30 µg/mL and 23.50 ± 1.70 µg/mL, respectively. Zeylenol **(2)** was selectively active towards inhibition of leukemic cancer cell (HL-60) with IC_50_ value of 11.65 ± 0.52 µg/mL. Acetylation of the hydroxyl groups of zeylenol **(2)** resulted in the elimination of its antileukemic activity for compounds **3** and **4** while weaker activities were also observed for the epoxide derivative **(5)** (IC_50_ 20.91 ± 1.28 µg/mL). As for zeylenol diacetate **(3)**, the anticancer property against HT-29 cell line (IC_50_ 6.73 ± 0.68 µg/mL) was better than its starting material [zeylenol (**2)**]. To the best of our knowledge, this is the first report of the cytotoxic activities of (+)-zeylenol **(2)** and its derivatives **(3-5)**. 

**Table 2:** Cytotoxic activity of compounds against HL-60, MCF-7, HT-29 and HeLa cells.

	IC_50_(μg/mL)			
Compounds	HL-60	MCF-7	HT-29	HeLa
**1**	24.22 ± 0.30	23.50 ± 1.70	-	-
**2**	11.65 ± 0.52	-	-	-
**3**	-	-	6.73 ± 0.68	-
**4**	-	-	-	-
**5**	20.91 ± 1.28	-	NT	-
*Positive control*				
Goniothalamin	1.23 ± 0.20			
5-Fluorouracil			4.20 ± 0.30	
Tamoxifen		3.12 ± 0.20		1.10 ± 0.21

*Notes*: HL-60 (Human Promyelocytic Leukemia), MCF-7 (Human Breast Cancer), HT-29 (Human Colon Cancer), HeLa (Human Cervical Leukemia). - : not active, IC_50_ > 30 μg/mL; NT: not tested. * Results are expressed as IC_50_ values (μg/mL) ± Standard deviation of three experiments performed in triplicate.

## 3. Experimental

### 3.1. General

Melting points were determined using a Barnstead Electrothermal IA 9100 series melting point equipment and were uncorrected. Optical rotations were measured on a JASCO P-2000 series polarimeter. UV spectra were obtained by using Shimadzu 1650 PC spectrophotometer. IR spectra were recorded using a Perkin Elmer FTIR spectrophotometer model Spectrum BX. The HRESIMS mass spectra were obtained from a Bruker Daltonics micrOTOF-Q ESI-Qq-TOF mass spectrometer. ^1^H-NMR and ^13^C-NMR spectra were recorded with a JEOL FT NMR spectrometer (500 MHz and 125 MHz for compound **1** and 400 MHz/100 MHz for other compounds) using TMS as internal standard. Silica gel (70-230 mesh, 230-400 mesh, Merck) was used for column chromatography. TLC was performed on silica gel plates (Merck DC-Alufolien 60 F_254_) and the spots were visualized under UV (254 nm, 366 nm) followed by spraying with 10% aqueous H_2_SO_4_ and further heating on a hot plate. 

### 3.2. Plant Material

Rhizomes of *Kaempferia angustifolia* Rosc. were collected from Yogyakarta, Indonesia in 2001 and identified by Sugeng Riyanto from Gadjah Mada University, Indonesia. A voucher specimen was deposited in the herbarium of the institution.

### 3.3. Extraction and Isolation

Rhizomes of *Kaempferia angustifolia* Rosc. were air-dried and ground into powder form. The plant sample (765 g) was extracted with methanol three times for three days each to give crude methanol extract which was then partitioned with hexane, water with methanol (10%) and chloroform (1 L each) to give hexane-soluble fraction (10.60 g) and chloroform-soluble fraction (42.50 g). The aqueous with methanol fraction was further discarded. The hexane extract (8.00 g) was subjected to vacuum column chromatography and eluted with mixtures of hexane, ethyl acetate and methanol to give 85 fractions (200 mL each) which were then combined according to their similar TLC profiles. Upon purification, (24*S*)-24-methyl-5α-lanosta-9(11),25-dien-3β-ol (40 mg), β-sitosterol (35 mg), 2′-hydroxy-4,4′,6′-trimethoxychalcone (7 mg), crotepoxide (10 mg) and β-sitosterol-3-*O*-β-D-glucopyranoside (8 mg) were obtained. Part of the chloroform extract (40.00 g) was fractionated in the similar manner to obtain 100 fractions (200 mL each) which were combined into major fractions based on their TLC profiles. 2′-Hydroxy-4,4′,6′-trimethoxychalcone (10 mg), boesenboxide (16 mg), crotepoxide (0.79 g), zeylenol (0.97 g) and kaempfolienol (abiet-8(14)-enepenta-6,7,9,11,13-ol, **1**, 20 mg) were obtained. 

### 3.4. Structural Modification of Zeylenol

Zeylenol (**2**, 0.10 g; 2.604 × 10^-4^ mole) was subjected to acetylation using acetic anhydride (1.0 mL; 1.06 × 10^-2^ mole) and pyridine (2 mL) with stirring for 2 hours at room temperature. The reaction was monitored by using thin-layer chromatography. Upon completion, the reaction crude product was poured into iced-water (10 mL) and extracted with dichloromethane (2 × 10 mL). The organic layer was then washed with 20% hydrochloric acid and further dried by adding anhydrous sodium sulphate. The crude product (0.17 g) was obtained after filtering off the drying agent and evaporating the solvent under reduced-pressure. Purification of the product using silica gel 9385 column chromatography was carried out. Eluent with a solvent system of hexane-ethyl acetate (70:30 → 60:40) gave zeylenol diacetate (**3**, 0.12 g; 99.3% yield).

Acetic anhydride (3.0 mL; 3.17 × 10^-2^ mole) was added to zeylenol (**2**, 0.10 g; 2.604 × 10^-4^ mole) in pyridine (4.0 mL) and the mixture refluxed for six hours. The reaction product was worked-up similarly to **3** to give crude product (0.20 g). The yellowish solid was then chromatographed on a silica gel column to yield a pale yellow oil which solidified at room temperature and was recrystallized with methanol to afford colourless needle-shaped crystals of zeylenol triacetate (**4**, 0.11 g; 77.5% yield).

Zeylenol (**2**, 0.05 g; 1.30 × 10^-4^ mole) was reacted with *meta*-chloroperbenzoic acid (MCPBA) (25 mg; 1.45 × 10^-4^ mole) in dichloromethane (10.0 mL) by reluxing for 24 hours. The solvent was evaporated from the reaction product under reduced-pressure and the crude mixture (94 mg) was purified by silica-gel column chromatography. Elution with hexane-ethyl acetate (70:30) afforded zeylenol epoxide (**5**, 12 mg; 23.0% yield).

### 3.5. Spectral Data

*Kaempfolienol* (**1**). Colourless needle-shaped crystals. m.p. 278-280 °C. [α]_D_ = + 1.8 ° (*c* 0.13, MeOH). UV (EtOH) λ_max _nm (log ε): 273 (2.70), 202 (4.06). IR (cm^-1^, KBr disc) ν_max _: 3,438, 2,934, 2,884, 1,628, 1,436, 1,384, 1,342, 1,018. For ^1^H- and ^13^C-NMR spectra, see Table 1. HR-ESIMS *m/z* (theoretical mass): 377.2746 ([M+Na]^+^, 377.2304), 355.3210 ([M+H]^+^, 355.2484).

*Zeylenol* (**2**). White amorphous solid. m.p. 108-110 °C (Lit. [4]: 125-126 °C). [α]_D_ = + 123.4 ° (*c* 0.05, CHCl_3_) [Lit.: [α]_D_ = + 121.6 ° (*c* 0.05, CHCl_3_)] [4]. UV (CHCl_3_) λ_max _nm (log ε): 275 (3.35), 241 (4.18). IR (cm^-1^, KBr disc) ν_max _: 3,470, 3,062, 2,912, 1,692 (C=O), 1,602, 1,586, 1,452, 1,374, 1,122, 1,072, 712. ^1^H-NMR (400 MHz, CD_3_OD): δ 8.03 (2H, *d*, *J* = 7.4 Hz, H-2″/6″), 7.99 (2H, *d*, *J* = 7.4 Hz, H-2′/6′), 7.56 (2H, *m*, H-4′/4″), 7.42 (4H, *m*, H-3′/5′, H-3″/5″), 5.94 (1H, *dd*, *J* = 10.1, 4.6 Hz, H-5), 5.81 (1H, *dd*, *J* = 10.1, 2.4 Hz, H-4), 5.69 (1H, *dd*, *J* = 6.4, 2.4 Hz, H-3), 4.68 (1H, *d*, *J* = 11.9 Hz, H-7a), 4.59 (1H, *d*, *J* = 11.9 Hz, H-7a), 4.34 (1H, br *d*, *J* = 4.6 Hz, H-6), 4.22 (1H, *d*, *J* = 6.4 Hz, H-2). ^13^C-NMR (100 MHz, CD_3_OD): δ 168.3 (7-OCO), 167.9 (3-OCO), 134.3 (C-4″), 134.1 (C-4′), 131.5 (C-1″), 131.4 (C-1′), 131.3 (C-5), 130.7 (C-2″/6″), 130.6 (C-2′/6′), 129.5 (C-3″/5″), 129.4 (C-3′/5′), 127.8 (C-4), 76.6 (C-1), 75.5 (C-3), 71.0 (C-2), 70.0 (C-6), 68.1 (C-7). HR-ESIMS *m/z* (theoretical mass): 407.1655 ([M+Na]^+^, 407.1107), 385.1801 ([M+H]^+^, 385.1287). 

*Zeylenol diacetate* (**3**). Colourless oil. [α]_D_ = + 24.8 ° (*c* 0.14, CHCl_3_) [Lit. [13]: [α]_D_ = + 17 ° (*c* 1.0, CHCl_3_)]. UV (CHCl_3_) λ_max _nm (log ε): 282 (3.60), 275 (3.67), 240 (4.52). IR (cm^-1^, NaCl disc) ν_max _: 3,464, 2,962, 1,728, 1,602, 1,448, 1,372, 1,264, 1,110, 712. ^1^H-NMR (400 MHz, CDCl_3_): δ 7.99 (4H, *m*, H-2′/6′, H-2″/6″), 7.56 (2H, *m*, H-4′/4″), 7.42 (4H, *m*, H-3’/5’, H-3″/5″), 6.01 (1H, *dd*, *J* = 10.1, 2.8 Hz, H-4), 5.95 (1H, *ddd*, *J* = 10.1, 4.6, 1.8 Hz, H-5), 5.88 (1H, *ddd*, *J* = 7.3, 2.8, 1.8 Hz, H-3), 5.71 (1H, *d*, *J* = 7.3 Hz, H-2), 5.51 (1H, *d*, *J* = 4.6 Hz, H-6), 4.65 (1H, *d*, *J* = 12.0 Hz, H-7a), 4.46 (1H, *d*, *J* = 12.0 Hz, H-7b), 3.16 (1H, s, 1-OH), 2.12 (3H, *s*, 6-OCOCH_3_), 2.09 (3H, *s*, 2-OCOCH_3_). ^13^C-NMR (100 MHz, CDCl_3_): δ 170.3 (2-OCO), 169.7 (6-OCO), 166.7 (7-OCO), 165.9 (3-OCO), 133.5 (C-4″), 133.3 (C-4′), 129.2 (C-5), 125.8 (C-4), 129.7 (C-2′/6′, C-2″/6″), 129.3 (C-1″), 129.0 (C-1′), 128.5 (C-3″/5″), 128.4 (C-3′/5′), 74.1 (C-1), 71.7 (C-2), 70.9 (C-3), 70.3 (C-6), 65.9 (C-7), 20.9 (2-OCOCH_3_), 20.8 (6-OCOCH_3_). HR-CIMS *m/z* (theoretical mass): 469.1497 ([M+H]^+^, required 469.1499).

*Zeylenol triacetate* (**4**). Colourless needle-shaped crystals. m.p. 150-151 °C (Lit. [13]: 158-159 °C). [α]_D_ = + 26.7 ° (*c* 0.30, CHCl_3_) [Lit. [13]: [α]_D_ = + 24 ° (*c* 0.6, CHCl_3_)]. UV (CHCl_3_) λ_max _nm (log ε): 283 (3.24), 275 (3.35), 242 (4.14). IR (cm^-1^, KBr disc) ν_max_: 3,062, 2,986, 2,954, 1,752, 1,726, 1,604, 1,454, 1,374, 1,104, 712. ^1^H-NMR (400 MHz, CDCl_3_): δ 8.03 (4H, *m*, H-2′/6′, H-2″/6″), 7.57 (2H, *m*, H-4′/4″), 7.47 (2H, *d*, *J* = 8.3 Hz, H-3″/5″), 7.43 (2H, *d*, *J* = 8.3 Hz, H-3′/5′), 6.19 (1H, br *d*, *J* = 4.6 Hz, H-6), 6.06 (1H, *dd*, *J* = 10.1, 2.8 Hz, H-4), 5.99 (1H, *d*, *J* = 8.3 Hz, H-2), 5.96 (1H, *ddd*, *J* = 10.1, 4.6, 1.8 Hz, H-5), 5.89 (1H, *ddd*, *J* = 8.3, 2.8, 1.8 Hz, H-3), 5.09 (1H, *d*, *J* = 12.0, H-7a), 4.82 (1H, *d*, *J* = 12.0, H-7b), 2.13 (3H, *s*, 1-OCOCH_3_), 2.03 (3H, *s*, 6-OCOCH_3_), 1.96 (3H, *s*, 2-OCOCH_3_). ^13^C- NMR (100 MHz, CDCl_3_): δ 169.9 (2-OCOCH_3_), 169.5 (6-OCOCH_3_), 169.2 (1-OCOCH_3_), 166.0 (7-OCO), 165.5 (3-OCO), 133.4 (C-4″), 133.3 (C-4′), 130.0 (C-5), 129.8 (C-2″/6″), 129.6 (C-2′/6′), 129.4 (C-1″), 129.2 (C-1′), 128.5 (C-3′/5′, C-3″/5″), 125.3 (C-4), 81.4 (C-1), 70.9 (C-3), 70.7 (C-2), 67.9 (C-6), 63.1 (C-7), 21.6 (1-OCOCH_3_), 20.8 (6-OCOCH_3_), 20.7 (2-OCOCH_3_). HR-CIMS *m/z* (% intensity): 511.1607 ([M+H]^+^, required 511.1604).

*Zeylenol epoxide* (**5**). Colourless needle-shaped crystals. m.p. 164-166 °C (Lit. [13]: 173-175 °C). [α]_D_ = + 61.4 ° (*c* 0.08, CHCl_3_) [Lit. [13]: [α]_D_ = + 68 ° (*c* 0.3, CHCl_3_)]. UV (CHCl_3_) λ_max _nm (log ε): 283 (3.14), 276 (3.23), 242 (4.05). IR (cm^-1^, KBr disc) ν_max _: 3,476, 2,924, 1,696, 1,600, 1,450, 1,279, 1,069, 709. ^1^H-NMR (400 MHz, CDCl_3_): δ 8.10 (2H, *d*, *J* = 8.3 Hz, H-2′/6′), 8.04 (2H, *d*, *J* = 8.3 Hz, H-2″/6″), 7.59 (2H, *d*, *J* = 8.3 Hz, H-4′/4″), 7.44 (4H, *t*, *J* = 8.3 Hz, H-3′/5′, H-3″/5″), 5.71 (1H, *dd*, *J* = 8.2, 1.8 Hz, H-3), 4.84 (1H, *d*, *J* = 11.9 Hz, H-7a), 4.58 (1H, *d*, *J* = 11.9 Hz, H-7b), 4.16 (1H, *d*, *J* = 4.6 Hz, H-6), 4.02 (1H, *d*, *J* = 8.2 Hz, H-2), 3.79 (1H, *dd*, *J* = 3.7, 1.8 Hz, H-4), 3.67 (1H, *dd*, *J* = 3.7, 4.6 Hz, H-5), 3.39 (1H, br *s*, OH), 2.96 (1H, br *s*, OH), 2.61 (1H, *d*, *J* = 8.2 Hz, 6-OH). ^13^C-NMR (100 MHz, CDCl_3_): δ 167.6 (7-OCO), 167.1 (3-OCO), 133.5 (C-4′/4″), 130.0 (C-2′/6′), 129.8 (C-2″/6″), 129.4 (C-1″), 129.3 (C-1′), 128.5 (C-3′/5′, C-3″/5″), 77.1 (C-1), 74.1 (C-3), 68.9 (C-2), 68.1 (C-6), 66.6 (C-7), 57.0 (C-4), 54.8 (C-5). HRFAB-MS *m/z* (theoretical mass): 401.1242 ([M+H]^+^, required 401.1236). 

### 3.6. Cytotoxicity Assays

Compounds were assayed for cytotoxic test against HL-60, MCF-7, HT-29 and HeLa cell lines using the MTT method [6]. Results were expressed as IC_50_ (μg/mL) ± standard deviation from three separate experiments, each performed in triplicate. 

## 4. Conclusions

From the rhizome extract of *Kaempferia angustifolia* Rosc., a new abietene diterpene identified as 5*S*,6*S*,7*S*,9*S*,10*S*,11*R*,13*S*-abiet-8(14)-enepenta-6,7,9,11,13-ol to which the trivial name kaempfolienol has been assigned was isolated. Cyclohexane derivatives, chalcone, triterpenes and glycoside were also obtained from the plant. Several derivatives of zeylenol were synthesized and the cytotoxic properties against some selected cancer cell lines of these compounds were studied. 
